# Mortalidade materna: protocolo de um estudo integrado à pesquisa *Nascer no Brasil II*

**DOI:** 10.1590/0102-311XPT107723

**Published:** 2024-05-17

**Authors:** Silvana Granado Nogueira da Gama, Sonia Azevedo Bittencourt, Mariza Miranda Theme, Maira Libertad Soligo Takemoto, Sônia Lansky, Paulo Germano de Frias, Barbara Vasques da Silva Ayres, Rosa Maria Soares Madeira Domingues, Marcos Augusto Bastos Dias, Ana Paula Esteves-Pereira, Marcos Nakamura-Pereira, Maria do Carmo Leal

**Affiliations:** 1 Escola Nacional de Saúde Pública Sergio Arouca, Fundação Oswaldo Cruz, Rio de Janeiro, Brasil.; 2 Levatrice Cursos, Rio de Janeiro, Brasil.; 3 Secretaria Municipal de Saúde de Belo Horizonte, Belo Horizonte, Brasil.; 4 Programa de Pós-graduação em Avaliação em Saúde, Instituto de Medicina Integral Prof. Fernando Figueira, Recife, Brasil.; 5 Instituto Nacional de Infectologia Evandro Chagas, Fundação Oswaldo Cruz, Rio de Janeiro, Brasil.; 6 Instituto Nacional de Saúde da Mulher, da Criança e do Adolescente Fernandes Figueira, Fundação Oswaldo Cruz, Rio de Janeiro, Brasil.

**Keywords:** Maternal Mortality, Risk Factors, COVID-19, Mortalidade Materna, Fatores de Risco, COVID-19, Mortalidad Materna, Factores de Riesgo, COVID-19

## Abstract

O *Estudo da Mortalidade Materna* conduz uma investigação hospitalar dos óbitos maternos ocorridos em 2020/2021 nas maternidades amostradas na pesquisa *Nascer no Brasil II*, com os seguintes objetivos: estimar o sub-registro da mortalidade materna e calcular um fator de correção e a razão de mortalidade materna (RMM) corrigida; validar as causas de mortalidade materna informadas na declaração de óbito (DO); e analisar os fatores associados à mortalidade materna. O *Nascer no Brasil II* inclui aproximadamente 24.255 puérperas distribuídas em 465 hospitais públicos, privados e mistos com ≥ 100 partos de nascidos vivos/ano nas cinco macrorregiões do país. Os dados do *Estudo da Mortalidade Materna* serão preenchidos utilizando o mesmo questionário do *Nascer no Brasil II*, a partir da consulta aos prontuários hospitalares. Obstetras treinados preencherão uma nova DO (DO refeita) a partir de análise independente desse questionário, comparando aos dados oficiais. A base de dados dos óbitos investigados será relacionada com os óbitos constantes no Sistema de Informações sobre Mortalidade do Ministério da Saúde, permitindo a estimativa do sub-registro e cálculo da RMM corrigida. Para o cálculo da confiabilidade das causas de morte, serão utilizados os testes kappa e kappa ajustado à prevalência com intervalo de 95% de confiança. Um estudo de caso-controle para estimar os fatores de risco para mortalidade materna será desenvolvido com os óbitos investigados (casos) e os controles obtidos na pesquisa *Nascer no Brasil II*, utilizando-se modelos de regressão logística múltipla condicional. Espera-se contribuir para a correção do sub-registro da mortalidade materna e para a melhor compreensão dos fatores determinantes da persistência de RMM elevada no Brasil.

## Abstract (en)

The *Maternal Mortality Study* conducts a hospital investigation of maternal deaths that occurred in 2020/2021 in the maternity hospitals sampled by the *Birth in Brazil II* survey, with the following objectives: estimate the maternal mortality underreporting; calculate a correction factor and the corrected (MMR); validate the causes of maternal mortality reported in the death certificate (DC); and analyze the factors associated with maternal mortality. The *Birth in Brazil II* includes approximately 24,250 puerperal women distributed in 465 public, private, and mixed hospitals with ≥ 100 live births/year in the five macroregions of Brazil. The *Maternal Mortality Study* data will be completed using the same *Birth in Brazil II* questionnaire, from the consultation of hospital records. Trained obstetricians will fill out a new DC (redone DC) from independent analysis of this questionnaire, comparing it to official data. The database of the investigated deaths will be related to the deaths listed in the Mortality Information System of the Brazilian Ministry of Health, allowing the estimation of underreporting and calculation of the corrected MMR. To calculate the reliability of the causes of death, the kappa test and prevalence-adjusted kappa with 95% confidence interval will be used. A case-control study to estimate the risk factors for maternal mortality will be developed with the investigated deaths (cases) and the controls obtained in the *Birth in Brazil II* survey, using conditional multiple logistic regression models. We expect this research to contribute to the correction of the underreporting of maternal mortality and to a better understanding of the determinants of the persistence of a high MMR in Brazil.

## Resumen

El *Estudio de Mortalidad Materna* evalúa las muertes maternas ocurridas en 2020-2021 en las muestras de maternidades del encuesta *Nacer en Brasil II* con los objetivos de estimar el subregistro de mortalidad materna y calcular el factor de corrección y la tasa de mortalidad materna corregida (TMM); validar las causas de mortalidad materna reportadas en el certificado de defunción (CD); y analizar los factores asociados a la mortalidad materna. La *Nacer en Brasil II* incluye aproximadamente 24.250 mujeres puerperales, distribuidas en 465 hospitales públicos, privados y mixtos con ≥ 100 nacidos vivos/año en las cinco macrorregiones de Brasil. Los datos de *Estudio de Mortalidad Materna* se completarán con la información del cuestionario *Nacer en Brasil II* a partir de una búsqueda de los registros médicos hospitalarios. Los obstetras capacitados completarán un nuevo CD (CD rehecho) desde un análisis independiente de este cuestionario, comparándolo con los datos oficiales. La base de datos de muertes investigadas se relacionará con las muertes que constan en el Sistema de Informaciones sobre la Mortalidad del Ministerio de Salud para permitir la estimación del subregistro y el cálculo de la TMM corregida. Para calcular la exactitud de las causas de muerte, se utilizarán las pruebas kappa y kappa ajustada a la prevalencia con un intervalo de 95% de confianza. Un estudio de casos y controles se aplicará para estimar los factores de riesgo de las mortalidad materna con las muertes investigadas (casos) y los controles obtenidos en el estudio *Nacer en Brasil II* utilizando modelos de regresión logística múltiple condicional. Se espera que este estudio pueda contribuir a la corrección del subregistro de la mortalidad materna y a una mejor comprensión de los determinantes de la persistencia de alta TMM en Brasil.

## Introdução

A mortalidade materna é reconhecidamente uma violação dos direitos humanos, sexuais e reprodutivos das mulheres. Trata-se de uma morte prematura, altamente evitável, que gera repercussões negativas à estrutura e à dinâmica familiar ^1^.

A magnitude da mortalidade materna, expressa pela razão de mortalidade materna (RMM), revela uma relação estreita e complexa entre as disparidades socioeconômicas e falhas na assistência à saúde da mulher e ao ciclo gravídico puerperal, e reflete o nível de reconhecimento da sociedade para com os direitos das mulheres ^1^. No Brasil, a RMM é um dos piores indicadores de saúde, permanecendo como um grande desafio a ser superado ^2^^,^^3^.

Segundo dados do Organização Mundial da Saúde (OMS), estima-se que, em 2020, morreram 287 mil mulheres por causas maternas no mundo, gerando uma RMM média de 223 óbitos maternos por 100 mil nascidos vivos, o que equivale a quase 800 mortes por dia. A grande maioria dessas mortes se concentra em países em desenvolvimento, apontando a imensa desigualdade na distribuição dos óbitos maternos, que chegam a alcançar uma RMM de 1.047 por 100 mil nascidos vivos na Nigéria, em contraponto à Noruega, com dois óbitos maternos por 100 mil nascidos vivos ^4^.

Na América Latina, houve pouco avanço na redução da RMM entre 2000 e 2020. Especificamente no período 2016 a 2020, primeiro quinquênio dos Objetivos do Desenvolvimento Sustentável (ODS) ^5^, houve incremento das mortes maternas na região ^4^. No Brasil, a queda da RMM, entre 1990 e 2019, foi de 39%, passando de 111,4 para 62,1 óbitos por 100 mil nascidos vivos ^6^. Ainda assim, não cumprimos o quinto Objetivo de Desenvolvimento do Milênio, de reduzir a RMM em 70% entre 1990 e 2015, e permanecemos distantes de alcançar a meta pactuada pelo governo brasileiro com os ODS, de atingir uma RMM de 30 por 100 mil nascidos vivos em 2030 ^7^.

Além disso, a emergência da COVID-19, declarada como pandemia pela OMS, desencadeou um expressivo acréscimo de óbitos de gestantes e puérperas ^8^^,^^9^^,^^10^, e o Brasil se tornou um dos países com maior número de óbitos maternos associados à infecção pelo SARS-CoV-2 do mundo ^11^^,^^12^. Dados do boletim do Observatório Obstétrico Brasileiro revelam 2.055 óbitos maternos em mulheres com COVID-19 registrados no país de 2020 a 2022, sendo 462 em 2020, 1.518 em 2021 e 75 em 2022 ^13^. Esse cenário expõe as fragilidades do cuidado obstétrico no país e impõe grandes mudanças a fim de que o Brasil atinja a meta dos ODS.

A COVID-19 mudou drasticamente a relação entre óbitos maternos diretos e indiretos no Brasil durante a pandemia. Dados oficiais revelam que o número de óbitos maternos pelas principais causas diretas (hipertensão, hemorragia, infecção puerperal e aborto) não mudou substancialmente durante a pandemia, enquanto as mortes por causas infecciosas e parasitárias aumentaram de 10 a 35 vezes em relação a 2019 ^14^^,^^15^. Em 2020, foram registrados oficialmente 1.965 óbitos maternos no Brasil, sendo que o próprio Ministério da Saúde corrige esse número para 2.039. Já no ano de 2021, foram registrados oficialmente 3.030 óbitos maternos no Brasil, elevando a RMM para 113 óbitos maternos por 100 mil nascidos vivos, sendo que aproximadamente metade desses óbitos foi decorrente da COVID-19 ^14^^,^^16^.

Apesar da cobertura quase universal do pré-natal e do parto hospitalar, a assistência de qualidade à gestação, ao parto/aborto e ao nascimento se constitui em um dos principais desafios do Sistema Único de Saúde (SUS). Problemas de gestão nas redes de atenção ao parto fazem com que mulheres com maior risco, residentes fora dos grandes centros, sejam mais expostas a complicações e ao óbito materno por maior dificuldade de acesso a serviços de saúde especializados, com estrutura apropriada, como unidade de terapia intensiva (UTI) ^17^, levando a demoras na oferta do cuidado apropriado. Por outro lado, a hipermedicalização da assistência ao parto e o excesso de cesarianas expõem as mulheres a maiores riscos de complicações, morbidade e mortalidade materna ^18^^,^^19^^,^^20^.

Quanto ao monitoramento da mortalidade materna, o país ainda apresenta problemas de subnotificação de óbitos e incorreções na classificação da causa básica de morte. Persistem erros na determinação do tipo de óbito, se materno ou não, além da incompletude de dados, que comprometem as análises sobre a assistência prestada e as condições de vida das mulheres que morreram na gravidez, parto, aborto ou puerpério, quadro que varia substancialmente entre macrorregiões e estados brasileiros ^7^^,^^20^. O comprometimento da compreensão dos determinantes da mortalidade materna prejudica a definição de prioridades, diretrizes e políticas de saúde e, por conseguinte, a efetividade das ações de prevenção e controle da ocorrência desses óbitos.

Desde 2004, com o lançamento do Pacto Nacional pela Redução da Mortalidade Materna e Neonatal, o Ministério da Saúde, apoiado por estados e municípios, tem adotado algumas estratégias para pautar a mortalidade materna como prioridade e ampliar a qualidade dos seus dados. Entre elas, o estabelecimento de metas relacionadas ao aumento da cobertura das informações de mortalidade, o desenvolvimento de painéis de monitoramento das informações e a regulamentação da vigilância dos óbitos maternos, de mulheres em idade fértil e de causas mal definidas ^21^^,^^22^.

Foram, ainda, estabelecidos fluxos e prazos para agilizar a investigação compulsória desses eventos, ficando a cargo do sistema de saúde local (municipal ou estadual) a investigação e a análise dos óbitos. Tais esforços contribuíram tanto para a maior captação e identificação de óbitos maternos quanto para a qualificar as informações, o que resultou na redução do fator de correção aplicado à RMM do Brasil de 1,16% em 2010 para 1,05% em 2019 ^22^.

Estudos nacionais que possibilitem revisão de dados sobre as mortes maternas são imprescindíveis para ampliar as potencialidades da vigilância e a capacidade de resposta do sistema de saúde perante o problema ^23^. Essas iniciativas contribuem para aumentar a acurácia dos dados sobre mortalidade materna e a confiabilidade das informações disponíveis no Sistema de Informações sobre Mortalidade (SIM) e identificar fatores associados à morte materna no país, particularmente das mortes por COVID-19.

Este artigo visa apresentar o protocolo do *Estudo da Mortalidade Materna*, aninhado ao *Nascer no Brasil II: Pesquisa Nacional sobre Aborto, Parto e Nascimento* (*Nascer no Brasil II*), que terá como principais objetivos: (1) estimar o sub-registro dos óbitos maternos e calcular um fator de correção e a RMM corrigida na amostra; (2) validar as causas do óbito materno; (3) estimar a RMM segundo as principais causas do óbito materno; (4) investigar a associação entre os fatores socioeconômicos, demográficos, obstétricos, de acesso aos serviços de saúde, das condições de assistência ao pré-natal, da estrutura da maternidade e dos processos e procedimentos na atenção hospitalar ao aborto, ao parto, ao nascimento e ao puerpério com a morte materna, com ênfase aos óbitos maternos por COVID-19.

## Método

O *Estudo da Mortalidade Materna* é um estudo retrospectivo aninhado à pesquisa *Nascer no Brasil II* que analisará todos os óbitos maternos ocorridos em 2020-2021 nos 465 hospitais participantes da pesquisa. O *Nascer no Brasil II* é um inquérito nacional de base hospitalar que apresenta diversos objetivos, incluindo estimar a prevalência de agravos e fatores de risco durante a gestação, avaliar a assistência pré-natal, ao parto e às perdas fetais e verificar a ocorrência de desfechos maternos e perinatais negativos e seus fatores associados. Em razão de a morte materna ser um evento raro e da possibilidade de não-inclusão de casos de morte materna no *Nascer no Brasil II* pela própria natureza do estudo, o *Estudo da Mortalidade Materna* foi desenhado como estudo retrospectivo aninhado ao *Nascer no Brasil II* para permitir investigar os óbitos maternos utilizando as mesmas variáveis e os mesmos métodos de coleta de dados.

### Pesquisa Nascer no Brasil II

O *Nascer no Brasil II*, conduzido entre 2021 e 2023, definiu como população de estudo o conjunto de mulheres internadas por motivo de parto ou perda fetal precoce (incluindo os casos de abortamento, interrupção legal da gestação, gestação ectópica e gravidez molar) nos hospitais amostrados. A amostra foi selecionada em dois estágios: o primeiro correspondeu aos hospitais e o segundo às puérperas. Todos os hospitais com 100 ou mais nascidos vivos/ano, segundo o Sistema de Informações sobre Nascidos Vivos (SINASC) de 2017, foram elegíveis para sorteio. A estratificação se deu segundo macrorregião do país (Norte, Nordeste, Sudeste, Sul, Centro-oeste); tipo de hospital (público/misto/privado); localização (capital e municípios localizados em Região Metropolitana/não Região Metropolitana) e tamanho (100-499 nascidos vivos/ano, ≥ 500 nascidos vivos/ano). A amostra total corresponde a aproximadamente 24.255 mulheres, sendo estimado 2.205 por motivo de aborto e 22.050 por motivo de parto, distribuídas em 465 hospitais. Maiores detalhes do plano amostral estão disponíveis ^24^.

Os critérios de elegibilidade para o *Nascer no Brasil II* foram: puérperas com nascido vivo, independentemente do peso e da idade gestacional, ou natimorto com idade gestacional ≥ 22 semanas ou peso ≥ 500g; e mulheres internadas com diagnóstico de aborto. Foram excluídas aquelas com dificuldades de comunicação por distúrbio mental grave; que não compreendem português; as surdas e as com gestação trigemelar ou mais. Para todas as mulheres incluídas no *Nascer no Brasil II*, são realizadas entrevistas no puerpério imediato; fotografados cartões de pré-natal e laudos de ultrassonografia, quando disponíveis, para posterior extração de dados; e coletados dados do prontuário hospitalar após a alta ou no 42º dia pós-parto ou aborto, em caso de internação prolongada da puérpera, ou no 28º dia de vida, no caso de internação prolongada do recém-nascido. Duas entrevistas telefônicas, aos dois e aos quatro meses após o parto ou perda, são realizadas para avaliar a utilização de serviços após a alta, morbidade materna e neonatal, aleitamento materno, desfechos psicossociais e satisfação com o atendimento.

Adicionalmente, para avaliar a estrutura e processos assistenciais dos serviços de obstetrícia e neonatologia, são entrevistados face a face os gestores das maternidades, coordenadores/chefes da Obstetrícia, da Neonatologia, da Epidemiologia e da Farmácia. Nessa etapa, é utilizado um instrumento desenvolvido com base na *Resolução de Diretoria Colegiada* (RDC) *nº 50* e nas Portarias do Ministério da Saúde, como as de *nº 1.071/2005* e *nº 2.048/2002*. Ele é composto por blocos sobre recursos humanos, organização e processo de trabalho, monitoramento do cuidado e de resultados da assistência, entre outros. Mais informações estão disponíveis em Leal et al. ^24^.

### População do Estudo da Mortalidade Materna

São considerados elegíveis para o *Estudo da Mortalidade Materna* todos os óbitos maternos ocorridos nas maternidades incluídas no *Nascer no Brasil II* no período de 1º de janeiro de 2020 a 31 de dezembro de 2021, seja durante a gestação, parto, perda ou no puerpério até 42 dias (incluídos os casos de óbito durante internações na gestação ou no puerpério, não vinculadas ao parto ou esvaziamento uterino).

Nos anos de 2020 e 2021, ocorreram, no Brasil, aproximadamente dois milhões de nascimentos vivos nos hospitais participantes do *Nascer no Brasil II*. Considerando a RMM de 58 óbitos maternos por 100 mil nascidos vivos em 2019 ^6^, estima-se identificar em torno de 1.200 óbitos maternos para esta análise.

### Identificação dos óbitos maternos e coleta de dados

Paralelamente à coleta de dados do *Nascer no Brasil II*, para identificar os óbitos maternos para a inclusão no *Estudo da Mortalidade Materna*, é solicitada ao gestor de cada hospital uma listagem nominal contendo todos os óbitos maternos ocorridos nos anos de 2020 e 2021 na instituição, bem como autorização para acesso aos prontuários desses casos. Os dados para o *Estudo da Mortalidade Materna* são, então, extraídos exclusivamente do prontuário hospitalar e da caderneta da gestante (quando estiver anexada ao prontuário), utilizando o mesmo instrumento de coleta de dados empregado na coleta de informações do prontuário materno do *Nascer no Brasil II*, pela mesma equipe de coletoras de dados que fazem a coleta de prontuários para as mulheres incluídas na amostra do *Nascer no Brasil II* em cada maternidade. A coleta de dados é realizada por meio de formulários eletrônicos no aplicativo REDCap (https://redcap.fiocruz.br/redcap/), que permite avaliar a consistência e controlar a qualidade dos dados coletados, diminuindo, assim, os erros de digitação e arquivamento. Além disso, o acesso online ao banco de dados permite o monitoramento em tempo real do trabalho de campo por uma equipe de supervisores.

As coletoras de dados são predominantemente enfermeiras com formação ou experiência em obstetrícia, treinadas especificamente para os procedimentos dos estudos *Nascer no Brasil II* e *Estudo da Mortalidade Materna*. Com vistas a esclarecer possíveis dúvidas da equipe de campo no preenchimento do questionário do prontuário hospitalar, foi constituído um grupo de profissionais com expertise em obstetrícia, denominado “orientadores de prontuário”, que ficam disponíveis via WhatsApp os sete dias da semana durante todo o período de coleta de dados. Mais detalhes sobre o programa de treinamento e as atividades de monitoramento da qualidade dos dados no *Nascer no Brasil II* e *Estudo da Mortalidade Materna* estão disponíveis em Leal et al. ^24^.

### Relacionamento SIM e Estudo da Mortalidade Materna

Para atender ao objetivo de estimar o sub-registro dos óbitos maternos, será feito o relacionamento dos óbitos identificados no *Estudo da Mortalidade Materna* com a base de dados nominal dos óbitos de mulheres em idade fértil ocorridos entre 1º de janeiro de 2020 a 31 de dezembro de 2021, registrados no SIM, por acesso concedido pela Secretaria de Vigilância em Saúde e Ambiente do Ministério da Saúde. Para o relacionamento serão utilizadas as seguintes variáveis: número da declaração de óbito (DO); o código da maternidade no Cadastro Nacional de Estabelecimento de Saúde (CNES); o primeiro e último nomes da falecida; data de nascimento; data da internação; e data do óbito. Por meio desse relacionamento, será possível identificar óbitos reclassificados após a investigação e disponíveis no SIM como óbitos maternos, bem como óbitos identificados como maternos pela maternidade, mas não informados no SIM como tal. Óbitos maternos encontrados no SIM sem identificação pelos hospitais serão informados às maternidades para localização dos prontuários e posterior resgate dos dados. Assim, será formada uma nova base do *Estudo da Mortalidade Materna* acrescida dos óbitos maternos identificados no SIM. Do mesmo modo, óbitos maternos não declarados no SIM serão informados às Secretarias Estaduais e à Secretaria de Vigilância em Saúde e Ambiente/Ministério da Saúde.

### Revisão das causas de morte

Para atender ao objetivo de validar as causas do óbito, para cada questionário de prontuário de óbito materno será preenchida nova DO (DO refeita), de forma independente, por dois médicos obstetras que desconhecem a DO original. Tendo como base as informações contidas no questionário de prontuário hospitalar do óbito materno e da caderneta da gestante, se disponível, cada médico obstetra realizará o preenchimento de uma nova DO com os seguintes campos: variáveis socioeconômicas e demográficas selecionadas e a certificação das causas de óbito - Parte I, linhas a, b, c e d, e Parte II, disponível no aplicativo REDCap. No caso de discordância entre o grande grupo de causas ou categorias da Classificação Internacional de Doenças, 10ª revisão (CID-10), ou no detalhamento/especificação do grupo de causa ou subcategorias da CID-10 entre os dois médicos, uma nova DO será preenchida por um terceiro médico obstetra treinado. A DO refeita será submetida à codificação das causas do óbito de forma independente por dois técnicos formados pelo Centro Brasileiro de Classificação de Doenças, segundo as regras da CID-10 ^25^.

Para garantia da qualidade dos dados, esses profissionais participaram de curso de atualização sobre o preenchimento da DO materna, de 20 horas, na modalidade à distância, coordenado pelo Instituto Nacional de Saúde da Mulher, Criança e Adolescente Fernandes Figueira (IFF) em parceria com Grupo de Pesquisa da Saúde da Mulher, da Criança e do Adolescente da Escola Nacional de Saúde Pública Sergio Arouca (ENSP), Fundação Oswaldo Cruz (Fiocruz). Foram abordados aspectos do processo assistencial, considerando o contexto e as circunstâncias dos óbitos maternos, segundo a metodologia de causa raiz, com a finalidade de qualificar e subsidiar a certificação das causas de óbito, alinhada à orientação geral para preenchimento da DO, com o sequenciamento correto de causas segundo a CID-10 ^25^.

### Variáveis

A variável de desfecho para o *Estudo da Mortalidade Materna* será: óbito materno ocorrido durante a internação na gestação, para parto ou esvaziamento uterino ou até o 42º dia pós-parto, aborto por causas de óbito materno - capítulo XV da CID-10.

As principais variáveis de exposição obtidas pelo instrumento de coleta de dados do prontuário (utilizado tanto para a *Nascer no Brasil II* quanto para o *Estudo da Mortalidade Materna*) compreendem:

(1) Demográficas e socioeconômicas: idade materna, raça/cor de pele, anos de estudo completos, situação conjugal, macrorregião de residência;

(2) Clínicas e obstétricas: antecedente de doença crônica, história obstétrica (número total de gestações, partos e abortos; cesariana anterior), desfechos negativos anteriores (baixo peso ao nascer, prematuridade, natimorto, neomorto);

(3) Gestação atual: número de consultas de pré-natal, grupo de Robson, tipo de parto, intercorrências na gestação atual;

(4) Atenção hospitalar ao parto, aborto ou relacionada à internação na gestação ou puerpério: dados da assistência recebida, demoras no recebimento de intervenções críticas, morbidade materna, causas de óbitos.

Com base no formulário de estrutura e processos assistenciais dos serviços de obstetrícia e neonatologia dos hospitais, serão analisadas, também, as seguintes variáveis: localização; nível de complexidade do hospital; volume dos partos; densidade e distribuição dos profissionais de saúde; campo de prática de ensino certificado pelo Ministério da Educação e Ministério da Saúde; acesso a equipamentos, insumos e o grau de implementação de boas práticas na assistência ao parto e nascimento.

### Análise dos dados

Na primeira etapa da análise (para atender aos objetivos 1 a 3 do *Estudo da Mortalidade Materna*), o fator de correção dos óbitos maternos será calculado por macrorregião pela razão entre os óbitos maternos informados ao SIM e os identificados no *Estudo da Mortalidade Materna*, sobre os óbitos informados no SIM. O processo de identificação dos óbitos maternos nas maternidades estudadas e da estimação dos fatores de correção permitirá generalizar o total de óbitos maternos para todos os hospitais brasileiros. Em seguida, será estimada a RMM hospitalar total e por causa de óbito da amostra das maternidades do *Nascer no Brasil II*: número de óbitos maternos dividido pelo número de nascidos vivos registrados no SINASC nos anos de 2020 e 2021 por 100 mil nascidos vivos. Nessa etapa, será realizada, também, a comparação entre a codificação original da causa básica de morte e outros dados da DO disponibilizados no SIM, com os dados da DO refeita. Será efetuada por meio do percentual de concordância, kappa, o kappa ajustado à prevalência e o grau de concordância classificado segundo Landis & Koch ^26^.

Na segunda etapa, será conduzida uma análise descritiva dos óbitos por características socioeconômicas, demográficas, clínicas, estrutura e processos assistenciais dos serviços de obstetrícia. As causas básicas e associadas serão analisadas considerando as causas obstétricas diretas e indiretas. Especial atenção será dada às mortes que tiveram como causa a COVID-19 e a atenção hospitalar recebida por essas mulheres. Serão analisados, ainda, segundo o agrupamento de causas da CID-Mortalidade Materna (CID-MM) ^27^. O delineamento amostral do estudo permitirá investigar mais profundamente esse conjunto de variáveis descrito segundo as macrorregiões e o tipo de financiamento.

Na terceira etapa da análise, para determinar os fatores associados aos óbitos maternos (sociodemográfico, obstétrico, comportamental, acesso aos serviços de saúde, estrutura e processos assistenciais dos serviços de obstetrícia), será realizado um estudo caso-controle, no qual o grupo de casos será composto por todos os óbitos maternos ocorridos nos hospitais da pesquisa *Nascer no Brasil II* em 2020 e 2021 e os controles serão selecionados de forma não pareada entre a amostra representativa de mulheres entrevistadas na pesquisa *Nascer no Brasil II* pós-parto ou pós-abortamento, cuja coleta ocorreu entre 2021 e 2024. Para minimizar o impacto da diferença temporal, será realizada uma calibração dos controles do *Nascer no Brasil II* para o período de ocorrência dos casos do *Estudo da Mortalidade Materna*, usando características sociodemográficas e obstétricas extraídas do SINASC.

Serão utilizados modelos de regressão logística múltipla não condicional com estimativa das razões de chance bruta e ajustada, considerando o nível de 95% de significância. Toda a análise estatística será realizada empregando procedimentos para amostras complexas, com ponderação e calibração dos dados e incorporação do efeito de desenho, com uso dos softwares SPSS 22.0 (https://www.ibm.com/) e R, versão 3.5.1 (http://www.r-project.org).

### Questões éticas

A pesquisa *Nascer no Brasil II*, incluindo o estudo aninhado da mortalidade materna, foi aprovado na Comissão Nacional de Ética em Pesquisa (CONEP, CAAE: 21633519.5.0000.5240), em 11 de março de 2020. No que diz respeito ao *Estudo da Mortalidade Materna* especificamente, por ser um estudo retrospectivo a partir de revisão de prontuários de casos de óbito materno, foi aprovada a dispensa da assinatura do Termo de Consentimento Livre e Esclarecido (TCLE), sendo o acesso aos prontuários autorizado pela unidade hospitalar.

## Discussão

Embora seja um dos eventos mais relevantes da saúde pública do Brasil, os estudos sobre mortalidade materna são, em sua maioria, descritivos, com foco na discussão dos métodos para melhoria da estimativa da mortalidade materna ou identificação da causa básica de óbito, sendo poucos os que se propõem a investigar os determinantes dos óbitos maternos, quase sempre restritos a municípios ou hospitais. Ademais, o uso da principal fonte de dados de mortalidade materna, o SIM, ainda é limitado devido à persistência da desigualdade nas coberturas, na qualidade dos dados de morte materna entre as macrorregiões e Unidades da Federação (UF) e na dificuldade de avaliar o perfil sociodemográfico dos óbitos maternos, em decorrência da limitada disponibilidade desses dados na DO ^6^.

Essas questões justificam um estudo mais amplo e abrangente, que se proponha a: investigar o contexto hospitalar em que ocorre a maioria dos óbitos maternos; estudar variáveis além das incluídas na DO; estimar fatores de correção macrorregional do número de óbitos maternos, permitindo o cálculo da RMM mais próxima da realidade; e identificar os determinantes das mortes maternas. Este será o primeiro estudo brasileiro sobre mortalidade materna com dados primários realizado em uma amostra com representatividade nacional e macrorregional, de hospitais públicos, mistos e privados, nas capitais dos estados, cidades das regiões metropolitanas e outros municípios.

Estudo prévio, conduzido em 2010 pela Rede Nacional de Vigilância de Morbidade Materna Grave, analisou 140 óbitos ocorridos em 27 hospitais ao longo de um ano, buscando identificar características demográficas e clínicas dos casos, assim como causas e demoras no acesso à assistência adequada ^28^. Nesse sentido, a proposta deste estudo avança na compreensão da complexidade da mortalidade materna no país ao fazer um censo das mortes maternas ocorridas nos hospitais da amostra do *Nascer no Brasil II*, garantindo representatividade das maternidades do Brasil, ao longo de 2020 e 2021, permitindo análises estatísticas robustas, alcançando um número estimado, inicialmente, de 1.200 óbitos. Adicionalmente, para o estudo de caso-controle, estarão disponíveis os dados completos de prontuários das puérperas entrevistadas no *Nascer no Brasil II*, obtidos das mesmas maternidades e utilizando os mesmos procedimentos padronizados de coleta de dados que aqueles empregados no *Estudo da Mortalidade Materna*.

As causas dos óbitos serão analisadas por meio do CID-MM ^27^, uma classificação proposta pela OMS, que agrupa as causas dos óbitos, utilizando os códigos da CID-10: grupo 1, gravidez que termina em aborto; grupo 2, causas hipertensivas na gravidez, parto e puerpério; grupo 3, hemorragia obstétrica; grupo 4, infecção relacionada à gravidez; grupo 5, outras complicações obstétricas; grupo 6, complicações de manejo não antecipadas; grupo 7, complicações não obstétricas. Essa classificação auxilia na identificação da causa do óbito, sua interpretação e análise ^27^.

Por ser o óbito materno um evento raro, optou-se por realizar análise sob a forma de caso-controle para investigar os fatores associados à morte materna, com os controles selecionados na mesma amostra de hospitais, minimizando vieses, já que as características da população atendida e o tratamento ofertado tendem a ser similares. Ainda que haja uma diferença temporal do momento de ocorrência dos casos e dos controles, não se espera que tenham ocorrido mudanças substanciais na característica das mulheres e da estrutura hospitalar nesse período. Para minimizar o impacto da temporalidade, uma calibração dos controles pode ser realizada a partir de dados do SINASC, a fim de verificar se houve mudança significativa nas características sociodemográficas e obstétricas das mulheres. Os resultados poderão ser generalizados para a maioria dos estabelecimentos de saúde que realizam parto e assistência ao aborto no país. O número expressivo de óbitos maternos que se antecipa identificar expandirá o poder do estudo, viabilizando as avaliações de exposições menos frequentes.

O uso do mesmo instrumento de coleta de dados de prontuário no *Estudo da Mortalidade Materna* e no *Nascer no Brasil II* permitirá o cálculo de diversos indicadores sociodemográficos, obstétricos e assistenciais relacionados com a morte materna, utilizando variáveis definidas e coletadas de forma padronizada, que não constam na DO. Além disso, a avaliação da estrutura e dos processos assistenciais dos serviços de obstetrícia e neonatologia dos hospitais da amostra possibilitará traçar um panorama mais abrangente da capacidade dos serviços prestados pelas maternidades ^24^.

Para reduzir a possibilidade de viés de informação originado pelo processo de coleta de dados não cego de casos e de controles, diferentes estratégias serão empregadas: uso do mesmo questionário para coleta de dados de prontuário hospitalar, de manual de instrução com a descrição dos procedimentos padronizados, somados ao treinamento sistemático dos coletores de campo e monitoramento contínuo da qualidade dos dados e do trabalho da equipe de coleta.

Vale destacar que o relacionamento probabilístico dos registros das bases do *Estudo da Mortalidade Materna*, após a investigação hospitalar de óbito materno, com a base do SIM, permitirá a identificação das mortes maternas não declaradas. A revisão dos prontuários e o preenchimento das causas de óbito na DO refeita por dois obstetras, de forma independente, marca um ponto de destaque desta pesquisa, conferindo maior acurácia na informação da causa básica e das causas múltiplas de morte. Esses dados fornecerão subsídios aos comitês de óbitos e ao SIM sobre as principais causas de óbito que apresentam erros de preenchimento.

Contudo, ainda permanecem lacunas a respeito dos óbitos de mulheres em idade fértil não investigados pelos comitês, que, no período de 2018 a 2020, corresponderam a cerca de 10% dos casos constantes no SIM, com variações importantes entre as macrorregiões brasileiras, de 3,1% na Região Sul até 14,1% no Nordeste do Brasil ^29^.

É possível que os óbitos ocorridos em domicílio, via pública ou estabelecimentos de saúde com menos de 100 nascidos vivos/ano, que entre 2018 e 2020 representavam menos de 10% dos óbitos maternos registrados no SIM, apresentem um perfil diferente das mulheres que tiveram acesso aos serviços hospitalares estudados ^29^. Ainda que essas situações sejam pouco frequentes, podem afetar as estimativas da mortalidade materna, por ser um evento raro.

Pela coincidência com os anos de pico da pandemia pode também haver, neste estudo, super ou sub-representação dos óbitos por COVID-19 em determinada UF, caso os hospitais de referência para esses casos na gestação e puerpério tenham sido ou não incluídos na amostra. Ademais, a inclusão de um hospital de referência para COVID-19 em um estado pode, eventualmente, significar perda seletiva de óbitos por causas obstétricas diretas que estavam sendo referenciadas para outra unidade não selecionada na amostra. Certamente, o número de mortes maternas indiretas nesses anos foi desproporcional à média de anos anteriores e isso deve ser ponderado para cálculo do fator de correção ^13^^,^^14^.

Em razão de o *Estudo da Mortalidade Materna* se basear exclusivamente em dados de prontuários hospitalares, muitas vezes com número significativo de variáveis não preenchidas, incompletas ou inconsistentes, o estudo poderá não refletir todas as lacunas assistenciais e barreiras enfrentadas pelas mulheres, em particular aquelas associadas à demora no acesso à assistência hospitalar. Antecipa-se, ainda, a possibilidade de ausência de informações obtidas por meio do cartão de pré-natal, caso não estejam disponíveis nos prontuários hospitalares. Algumas estratégias serão adotadas para contornar esse problema, como a imputação múltipla de dados com equações encadeadas. Ainda assim, a fonte primária de dados apresenta vantagens quanto à completude de dados em relação às fontes secundárias, usualmente utilizadas para análise do óbito materno nos estudos brasileiros.

Estudos dessa natureza podem contribuir com a vigilância e monitoramento da mortalidade materna ao destacar a relevância de dados completos e precisos e o estabelecimento de referenciais objetivos para ampliar a “capacidade de resposta” do sistema de saúde, à medida que demonstra as necessidades de formulação e implementação de recomendações alinhadas aos resultados encontrados. A integração contínua da vigilância à capacidade de resposta construirá as bases para controlar futuras mortes maternas evitáveis.

Considerando a elevada frequência de desfechos maternos negativos no país, espera-se que os resultados deste estudo forneçam informações relevantes para a elaboração de políticas públicas nacionais e locais, visando assegurar a segurança da parturiente e a ampliação do acesso às boas práticas na assistência à mulher na gestação, trabalho de parto, parto e aborto. Adicionalmente, poderá ampliar a satisfação de mulheres e profissionais de saúde com a atenção ao parto, às perdas fetais precoces e às intercorrências clínicas e obstétricas da gestação ao puerpério.
